# Developing Requirements for a Digital Self-Care Intervention for Adults With Heart Failure: Qualitative Workshop Study

**DOI:** 10.2196/72589

**Published:** 2025-08-25

**Authors:** Rebecca Nourse, Lars Kayser, Ralph Maddison

**Affiliations:** 1 Institute for Physical Activity and Nutrition, School of Exercise and Nutrition Sciences, Deakin University, 221 Burwood Highway, Burwood, 3125, Australia, 61 0392446218; 2 Department of Public Health, University of Copenhagen, Copenhagen, Denmark

**Keywords:** self-care, heart failure, behavior change, digital health, co-design, user-centered design, Behavior Change Wheel

## Abstract

**Background:**

Heart failure is a complex syndrome that requires long-term management, including self-care, to prevent decompensation and hospitalization. Although a range of interventions exists, evidence supporting their effectiveness remains limited, prompting calls for more theory-informed and user-centered approaches. The rapid advancement of mobile and digital technologies offers new opportunities to improve self-care, particularly when interventions are grounded in behavioral theory and shaped by user input.

**Objective:**

This study aimed to define user-centered, theory-informed requirements for a digital intervention to support self-care among people with heart failure. We combined the Behavior Change Wheel (BCW) with user-centered design (UCD) to explore self-care barriers and generate actionable intervention requirements.

**Methods:**

A qualitative study was conducted, involving 4 workshops with people with heart failure (n=16) and informal caregivers (n=4) across metropolitan and regional Australia. Guided by UCD principles, the workshops explored self-care barriers and elicited ideas for a digital intervention. Barriers were coded using the capability, opportunity, motivation, and behavior (COM-B) model and the Theoretical Domains Framework to identify behavioral determinants and user needs. Ideas and preferences for the intervention were analyzed using requirements analysis and affinity mapping to generate themes describing intervention components (“what” the system should do) and user requirements (“how” it should operate). Intervention components were then mapped to relevant BCW intervention functions.

**Results:**

Participants identified self-care barriers across all 3 COM-B components and 11 of 14 Theoretical Domains Framework domains, including barriers related to capability (eg, lack of knowledge and forgetfulness), opportunity (eg, busy lifestyle and limited access to resources), and motivation (eg, emotional burden and lack of confidence). These were translated into 28 distinct user needs. From participants’ ideas, 6 themes relating to intervention components were identified: education, monitoring and feedback, social connection and support, psychological and emotional support, planning and preparing, and health care support. These components mapped to 7 BCW functions: education, persuasion, incentivization, training, environmental restructuring, modeling, and enablement. Additionally, 6 user requirement themes were developed: physical design, accessibility and usability, personalization and control, engagement and user experience, support and implementation, and integration and system organization.

**Conclusions:**

This study demonstrates the value of integrating UCD with the BCW to develop intervention requirements that are both user-centered and theoretically grounded. By exploring both what the intervention should do and how it should do it, we identified actionable requirements that bridge the gap between understanding behavior and developing effective solutions. Future work can focus on translating these requirements into prototype interventions and evaluating their feasibility, acceptability, and effectiveness.

## Introduction

### Heart Failure and Self-Care

Heart failure is a complex syndrome that requires long-term management to maintain and manage health [1]. Globally, heart failure remains a leading cause of morbidity and hospitalization, which results in reduced quality of life and life expectancy [2,3]. In Australia, it has been shown that hospitalization due to acute exacerbation of heart failure strongly predicts poor outcomes for those with the condition, equating to a 50% reduction in remaining life expectancy [4]. However, in this setting, up to 63% of hospital admissions are avoidable with appropriate community support and self-care [5], highlighting the importance of effective self-care interventions.

Self-care refers to the activities and behaviors undertaken to maintain, monitor, and manage health and well-being [6,7]. These activities include, for example, maintaining optimal nutrition, taking prescribed medications, managing psychological status, monitoring signs and symptoms, titrating medications, and contacting a health care practitioner in response to symptoms [8]. Previous studies have reported that people with heart failure often have insufficient self-care practices. For example, they may struggle to recognize their symptoms and, as a result, delay reporting to health care practitioners [9,10]. Additionally, up to 60% of people with heart failure do not take their medications as prescribed, and up to 80% do not follow recommended lifestyle guidance [11,12]. Consequently, numerous interventions to support self-care among people with heart failure have been developed.

Yet, a recent systematic review and meta-analysis found that despite the multitude of interventions available to support self-care among people with heart failure, evidence supporting their effectiveness in improving outcomes remains limited [13]. According to Riegel et al [14], self-care interventions should promote change across the range of self-care behaviors an individual needs to engage in and improve problem-solving and decision-making skills. To date, few interventions have comprehensively addressed these elements [15,16]. However, the rapid advancement of mobile and digital technologies has opened new avenues for developing digital health interventions to facilitate self-care for people with heart failure [17,18].

### The Potential for Digital Health Interventions in Heart Failure Self-Care

Digital health refers to the use of technology to improve health and well-being, including the enhancement of health care provision through the intelligent processing of health data [19]. As such, digital health is an overarching term that encompasses related concepts such as mobile health, telehealth, and clinical information systems [20]. Digital health has been increasingly applied to the prevention, detection, and management of heart failure [18,21] through tools such as telemonitoring, teleconsultations, wearable sensors, and mobile apps [18,21-23]. Advances in unobtrusive data collection and machine learning offer opportunities for designing self-care interventions. These approaches can support personalization of intervention content and delivery modes based on individual needs and contexts [16].

Personalization is of particular importance in heart failure, as self-care varies between and within individuals—from the combination of self-care behaviors, ways of performing a specific behavior (eg, physical activity can be performed by walking, cycling, or swimming), and objectives within a behavior (eg, target heart rate value). These variations in self-care between and within individuals result from factors such as health status and trajectory (eg, recent decompensation and comorbidities), current care plan (eg, medications and dosages), socioenvironmental situation (eg, social influence and weather), and personal preferences and values (eg, likes and dislikes) [17,24].

### Approaches to Intervention Development

As described earlier, heart failure self-care interventions require multiple components to address the diverse support needs of this population. Consequently, these interventions are considered complex interventions, which typically include multiple components, or target multiple behaviors, or require skills and expertise from those delivering and receiving them or are flexible in their approach [25].

The Medical Research Council guidance on developing and evaluating complex interventions advises selecting a framework to guide the intervention development process and base the intervention design on evidence and theory [25]. Numerous frameworks exist, including the Behavior Change Wheel (BCW), a theoretically grounded framework for intervention development [26,27], and user-centered design (UCD), which emphasizes the involvement of end users throughout the design process [28].

The BCW has been widely used to develop interventions targeting a variety of health behaviors, including physical activity and medication taking in people with chronic conditions [29-31]. In the context of heart failure self-care, one study applied the BCW to generate a set of evidence-based intervention features from clinical guidelines and literature, providing a foundation for the design of theory-based interventions [32]. However, as has been highlighted by the author of the aforementioned study and many others, interventions should also be adapted to address the local context, experiences, and needs of their intended users [32,33]. Involving end users in the design process can improve intervention acceptability [34,35]—a goal central to UCD [28]. Combining the BCW with UCD principles could result in interventions that are both theoretically informed and adapted to the real-world contexts of users.

### Aim

The aim of this study was to define requirements for a digital intervention to support self-care in people with heart failure. By combining the BCW and UCD approaches, we sought to identify self-care barriers, explore potential solutions, and translate these insights into theory-informed, user-centered intervention requirements. These findings contribute to the development of a digital health intervention intended to improve self-care among people with heart failure, potentially improving health outcomes and quality of life for this population [36].

## Methods

### Study Design

This study contributed to a larger research project, SmartHeart, which aimed to develop a digital health intervention to support people with heart failure in managing their health and improving key outcomes [36]. The proposed intervention included elements for both people with heart failure and health care practitioners and planned to integrate remote patient monitoring with behavioral support by leveraging digital health, including devices for data collection (eg, wearables and smart scales) and algorithms for personalization [36]. SmartHeart adopted a theory-based, user-centered approach to intervention design and development [37].

This paper reports on a qualitative study involving 4 workshops with people with heart failure and caregivers. The goal was to develop a set of user-centered, theory-informed intervention requirements for the patient-facing elements of the intervention. Requirements were defined as the criteria and functions an intervention must have to address user needs [38,39]. A key part of UCD, these requirements can be used by multidisciplinary teams (including the users themselves) to design effective, usable solutions [39]. Workshops were chosen because they facilitate collaborative learning, data creation, problem-solving, and innovation related to a specific issue [40], aligning with UCD principles of involving users in the design.

To ensure that the requirements were theory-informed, as well as user-centered, we applied the first two stages of the BCW: (1) BCW stage 1: Understanding the behavior—uses the capability, opportunity, motivation, and behavior (COM-B) model [26] to identify behavioral determinants that may contribute, positively or negatively, to the performance of a target behavior [26]. These behavioral determinants can be explored in further detail using the Theoretical Domains Framework (TDF), which is an extension of COM-B and provides 14 additional domains corresponding to the COM-B model [41]. (2) BCW stage 2: Identifying intervention options—links the barriers identified in step 1 to intervention functions that change behavior [26,27]. The BCW intervention functions are education, persuasion, incentivization, coercion, training, restriction, modeling, enablement, and environmental restructuring [26,27].

### Participants and Recruitment

Eligible participants were adults (18 years and older) with heart failure, living in the community (ie, at home), and able to communicate in English. We excluded individuals with severe heart failure (New York Heart Association IV), recent myocardial infarction, unstable angina, referral to an advanced heart failure transplant unit, and with severe chronic pulmonary disease (ie, requiring home oxygen). Those living in a long-term care establishment (eg, a care home) or unable to participate fully in the study for other reasons (eg, dementia) were also excluded. Caregivers of participating people with heart failure were also invited to take part.

Participants were recruited using purposive sampling from 4 hospital sites in Australia where the research team had existing relationships: 1 metropolitan site in Queensland and 3 sites in Victoria (1 metropolitan and 2 regional). At each site, a heart failure nurse identified eligible patients during routine outpatient clinic appointments and provided a brief description of the study. With permission, contact details of interested patients were forwarded to the research team, who then contacted them by telephone to discuss the study. Additionally, individuals who had participated in a previous interview study and consented to be recontacted for future research were invited by email. Participants were also offered the opportunity to invite a caregiver. This sought to gain a range of perspectives and ensured that participants had support available to them if required. In alignment with recommendations for qualitative research, we aimed to recruit between 20 and 32 participants in total, equating to 4‐8 participants at each of the 4 workshops [42].

### Setting

Workshops were held between March and August 2022, either in-person or online. In-person workshops were held in neutral, accessible locations: an education room attached to a local hospital and a conference room at a golf course. Online workshops were hosted on Zoom (Zoom Video Communications, Inc). These locations were chosen so that participants felt at ease and were able to share as readily as possible [43]. Participants were able to choose their preferred participation format in order to accommodate their preferences and geographical and health constraints. This was particularly important because the research was conducted shortly after the COVID-19 social distancing restrictions were lifted.

### Research Team and Reflexivity

Workshops were conducted by 2 female facilitators (RN and HT). Apart from the facilitators and the participants, no further persons were present during the sessions. The lead facilitator (RN), who also led the data analysis, had an MSc degree and was undertaking a PhD degree on digital health and self-care in heart failure at the time of the study. She had experience in public health and qualitative research. The second facilitator had an MSc in nutrition and prior experience with research interviews and group facilitation in health settings. Two further researchers each had clinical experience and supported data analysis and interpretation in discussion with the lead researcher (RN). One participant had previously taken part in an interview with the second researcher as part of an earlier phase of the SmartHeart project; no other participants had prior relationships with the researchers.

### Data Collection Procedures

**Figure 1.**
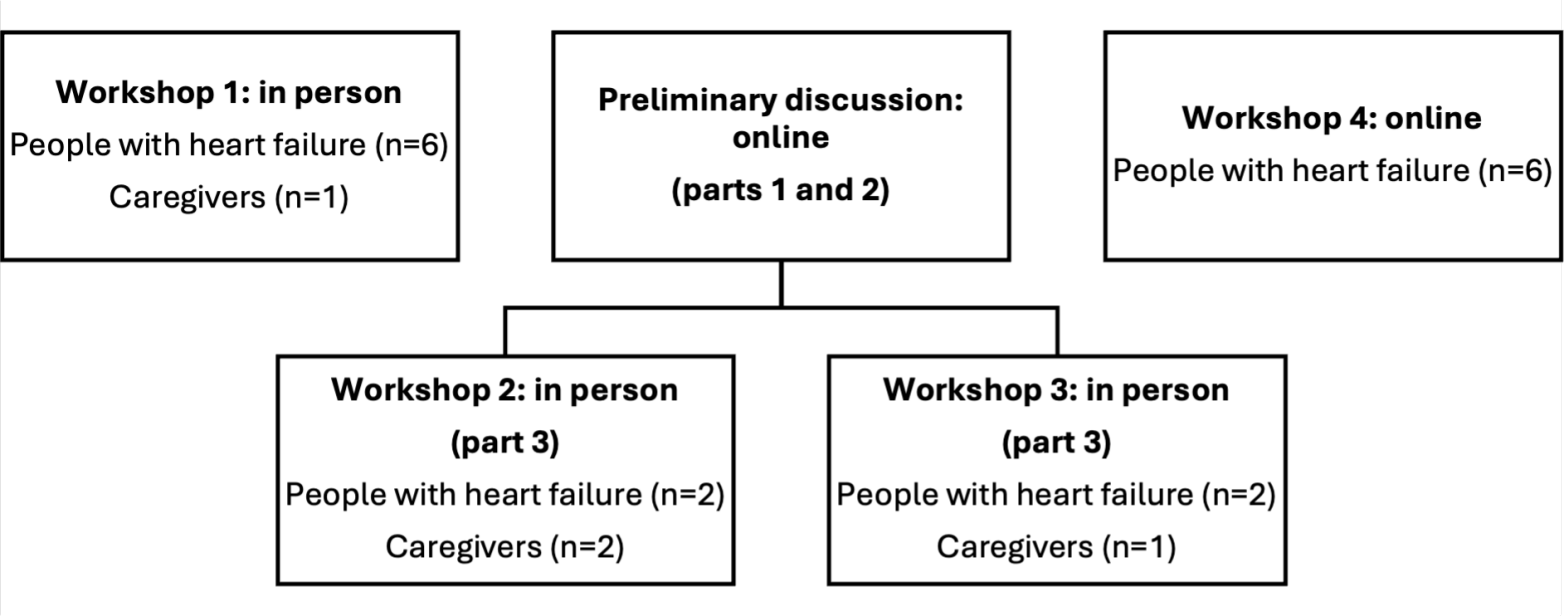
Overview of participant flow and workshop structure for identifying user requirements for a digital health self-care intervention for heart failure.

After consenting to participate, participants were asked to provide basic demographic information through an online form hosted on the QualtricsXM web-based software platform (SAP America Inc) and workshops were scheduled for a mutually convenient time. Sessions lasted between 1 and 4 hours, including time for breaks and refreshments during the longer in-person workshops. Two researchers facilitated the workshops, which included (1) an introduction, (2) a group discussion on self-care barriers, and (3) activities to elicit participant ideas and perspectives on potential features for the intervention. Workshops 2 and 3 were conducted in 2 parts: the first half online (covering the introduction and discussion on barriers) and the second half in-person (covering the idea generation activities). This structure was implemented following participant feedback from workshop 1, explained in more detail later. An overview of workshops is shown in Figure 1.

At the start of each workshop, the facilitators introduced themselves and their backgrounds. The introduction provided participants with a description of the SmartHeart research project, a high-level overview of the proposed intervention, and the aims of the session. This ensured a shared understanding of the purpose of the research. The discussion on barriers was participant-driven, with facilitators using prompts structured according to the TDF to stimulate additional or deeper exploration [41]. This ensured that issues relevant to the participants were raised and maintained a natural flow in the discussion [44]. To elicit participants’ ideas and perspectives on potential features for the intervention, we used different explorative and creative activities in each workshop:

- Workshop 1: Participants individually wrote down as many ideas for solutions as possible before sharing them with the group. In subgroups, they discussed their ideas together and sketched their ideas for intervention concepts. This activity was inspired by paper prototyping [45]. Each paper prototype was presented back to the main group and discussed.
- Workshops 2 and 3: Icon cards depicting everyday objects were provided as stimulus material. The cards contained objects identified during the research group’s previous work using wearable camera images [46]. When discussing solutions to each barrier, participants selected relevant cards and explored how they could be integrated into intervention designs.
- Workshop 4: A summary of the ideas generated in the previous workshops was shared with participants for feedback and further ideation. Inviting feedback on initial ideas both provided deeper insights and introduced an element of challenge to come up with new ones.

Throughout each workshop, participants were encouraged to write or draw their ideas on paper, which was then placed in a centrally visible location. This enabled participants to express their opinions in multiple ways and documented progress. Facilitators and caregivers assisted participants who required help. Both facilitators took notes during each workshop, with the second facilitator taking more extensive notes. Participant-created materials were also photographed for later analysis. Web-based workshops were audio-recorded.

At the end of each workshop, participants completed feedback forms rating their experience from 1 (did not enjoy) to 10 (really enjoyed) and answering open-ended questions about what they enjoyed, did not enjoy, and suggestions for improvement. For the in-person workshops (workshops 1-3), a feedback form was handed to participants at the end, and web-based workshop (workshop 4) participants were emailed with the same questions. The feedback was used to improve subsequent workshops. The feedback was also analyzed once all workshops had concluded (this is provided in the “Results” section).

Feedback from the first workshop indicated that participants wanted additional information to stimulate ideas. The facilitators noted that participants focused more on sharing their experiences than potential solutions. Therefore, before workshops 2 and 3, participants were invited to a 1-hour discussion session on Zoom to discuss their self-care experiences, focusing on barriers and facilitators. At the end of the web-based discussion, the activities for the in-person portion of the workshop were introduced and provided by email afterward. This gave participants more time to reflect on the topic and generate ideas. The icon cards were also introduced to stimulate ideas. Workshop 4 was held entirely on the web as participants were recruited from a range of locations, and there was no mutually convenient location to have an in-person workshop. Before this workshop, participants were emailed a summary of the findings to date, the aims of the workshop, and a list of discussion points for their reflection before the workshop if they wished.

### Data Analysis

#### Overview

Data analysis followed a systematic approach integrating the BCW and requirements analysis [39], a UCD method, to derive self-care barriers and intervention requirements. The analysis process is reported linearly, but in practice, we took an iterative approach whereby we revisited steps as new information was found. To prepare the data for analysis, the first author compiled and summarized material from each workshop including facilitator notes, participant notes and sketches, and audio recordings. These summaries captured participants’ self-care barriers, ideas for solutions, and perspectives on proposed ideas. The supporting facilitator reviewed the summaries for accuracy, and discrepancies were resolved through discussion. While the summaries were not returned to participants for comment or correction, participants in workshop 4 were presented with findings from earlier workshops and invited to reflect upon them—providing informal feedback on the analysis to that point. Salient participant quotes illustrating the findings were transcribed from the audio recordings. Data were organized using a combination of paper sticky notes and Microsoft Excel. One researcher (RN) led the analysis and discussed the findings with other members of the research team.

#### Self-Care Barriers and User Needs

Self-care barriers were coded using the COM-B model [26] and the TDF [41]. The coding process involved identifying specific barriers mentioned by participants and mapping these to COM-B components (capability, opportunity, and motivation) and corresponding TDF domains. For example, when participants described difficulty remembering medication, these statements were coded under the “psychological capability” COM-B component and the “memory, attention, and decision processes” TDF domain.

The findings were cross-checked against existing literature to support the interpretation of the data [47,48]. By workshop 4, no further self-care barriers were raised by participants. Each barrier was then translated into a user need. For example, the barrier “difficulty in interpreting symptoms and attributing their causes” was translated into “people with heart failure need to interpret symptoms and their causes.”

#### Intervention Requirements

For intervention requirements, we followed the requirements analysis process outlined by Van Velsen et al [39]. Each idea or perspective on a proposed solution was first summarized as an attribute (a higher-level summary). Then, attributes were grouped using affinity mapping, an approach used in UCD methodologies to visually organize qualitative data by grouping based on their meaning and relationships [49,50]. During this process, 2 categories of themes emerged: (1) themes pertaining to potential intervention components (the “what” requirements) and (2) themes describing user requirements (the “how” requirements). By Workshop 4, participants’ ideas and perspectives were captured by the themes generated. The themes pertaining to potential intervention components (the “what” requirements) were coded using the BCW intervention functions (education, persuasion, incentivization, coercion, training, restriction, modeling, enablement, and environmental restructuring) [26,27]. For example, when participants suggested medication reminders to address forgetfulness, this was classified under the “psychological and emotional support” component, which aligned with the “enablement” intervention function.

#### Participant Characteristics and Feedback

Participant characteristics were summarized using descriptive statistics. Data from feedback forms were summarized using descriptive statistics (quantitative data) and thematic analysis (qualitative data) [51].

### Ethical Considerations

This study received ethical approval from the Royal Melbourne Health Human Research Ethics Committee (reference number HREC/76317/MH-2021) and the Deakin University Human Research Ethics Committee (reference number DUHREC 2021‐440). All participants were given information about the study (eg, aims, eligibility, risks and benefits of participation, data processing and storage, and right to withdraw). The information contained contact details for the research team and advice to contact the researchers with any questions about the study. Informed consent was obtained via an online form hosted on the QualtricsXM web-based software platform (SAP America Inc), where participants were asked to review the study information and confirm their participation. Study data were deidentified. Identifiable data and deidentifiable data were stored separately on a secure password-protected server that only the research team could access. Each participant received a AU $100 (approximately US $60) voucher to recognize their time commitment and expenses.

## Results

### Participant Characteristics

A total of 20 people contributed to this study: people with heart failure (n=16) and informal caregivers (n=4). An additional 3 people with heart failure consented to participate in the study but could not attend a workshop due to ill health. Participants with heart failure had a median age of 66.0 years (IQR 56-73.5 years), with 56.3% (9/16) being female. Most (10/16, 62.5%) had university or college education. The majority (11/16, 68.8%) were not working at the time of the study, and 87.5% (14/16) reported having comorbidities alongside heart failure. Caregivers (n=4) had a median age of 43.5 years (IQR 31.5-60.5 years), with equal gender distribution (50% female and 50% male). Table 1 shows detailed participant characteristics.

**Table 1.** Demographic and clinical characteristics of study participants (N=20) who participated in workshops to identify user requirements for a digital health self-care intervention for heart failure.

Participant characteristics	People with heart failure (n=16)	Caregivers (n=4)
Age (years), median (IQR)	66.0 (56-73.5)	43.5 (31.5-60.5)
Sex, n (%)		
Female	9 (56.3)	2 (50.0)
Male	7 (43.8)	2 (50.0)
Education, n (%)		
University/college	10 (62.5)	3 (75.0)
High school	3 (18.8)	1 (25.0)
Some high school	2 (12.5)	0 (0.0)
Not reported	1 (6.3)	0 (0.0)
Living status, n (%)		
With 2 or more	4 (25.0)	0 (0.0)
With 1 other	10 (62.5)	4 (100.0)
Alone	2 (12.5)	0 (0.0)
Employment status, n (%)		
FT^a^	4 (25.0)	2 (50.0)
PT^b^	1 (6.3)	1 (25.0)
Not working	11 (68.8)	1 (25.0)
Years with heart failure, n (%)		
<1	2 (12.5)	N/A^c^
1‐3	7 (43.8)	N/A
4‐10	4 (25.0)	N/A
>10	2 (12.5)	N/A
Not reported	1 (6.3)	N/A
Comorbidities, n (%)		
Yes	14 (87.5)	N/A
No	2 (12.5)	N/A
Location, n (%)		
Metro	8 (68.8)	3 (75.0)
Regional	5 (31.2)	1 (25.0)

a FT: full time.

b PT: part time.

c N/A: not applicable.

While we did not formally collect information about experience with technology, discussions with participants during the workshops evidenced that all participants had some experience with technology, ranging from infrequent to frequent use and low to high confidence in using digital devices.

### Participant Feedback

Workshop feedback was returned by 19 of 20 participants (1 participant left before the end of an in-person workshop and was not followed up for their feedback afterward). Workshop feedback was predominantly positive, with participants rating their experience at an average of 9.3 out of 10 (range 6‐10). Most participants (n=13) rated their overall experience at the workshop at the maximum score of 10 (really enjoyed). Thematic analysis of the open-ended responses found that participants enjoyed meeting other people with similar experiences, exchanging ideas and stories, feeling heard, reading materials before the workshop, and contributing to improvements for heart failure care. However, some participants reported that it was difficult to let their thoughts flow as they felt nervous, and some thought that other people in the group spent too much time talking about their personal story. Participants’ suggestions for improving the workshops included longer discussion time on more topics, a short video with a practical demonstration of the intervention, physical examples of the intervention, and more direct questions and answers. Some of these suggestions were incorporated into the subsequent workshops as described in the “Methods” section and can be used to inform future intervention development workshops.

### Main Findings

#### Self-Care Barriers and User Needs

Analysis of self-care barriers revealed self-care barriers in all 3 COM-B domains (capability, opportunity, and motivation) and 11 of 14 TDF domains. The 3 TDF domains not represented in the data were reinforcement, optimism, and beliefs about consequences. The complete behavioral diagnosis, including illustrative quotes, is shown in Multimedia Appendix 1.

Participants reported barriers related to capability (eg, lack of knowledge and forgetfulness), opportunity (eg, busy lifestyle and limited access to resources), and motivation (eg, emotional burden and lack of confidence). For example, one participant explained: “Weight varies, I’ve been told—if my weight varies by 2 kgs, get in touch with doctor but it’s impossible to get in touch with doctor” (Workshop 2). This was coded under COM-B as “physical opportunity” and the TDF as “environmental context and resources.”

Translation of these barriers into user needs resulted in 28 user needs that an intervention might address to support self-care, such as: people with heart failure need to plan self-care tasks in advance, people with heart failure need clear and consistent advice on self-care, and people with heart failure need to have the confidence to perform self-care.

#### Intervention Components (“What” Requirements)

Analysis of participants’ ideas for the intervention resulted in the production of 6 themes describing intervention components—“what” an intervention should provide to help overcome barriers to self-care. Table 2 shows each intervention component theme, the specific participant-derived requirements associated with each, and the aligned BCW intervention functions.

**Table 2.** Intervention component themes, participant-derived requirements, and aligned Behavior Change Wheel intervention functions for a digital health self-care intervention for heart failure.

Intervention component theme	Requirements derived from participants’ ideas	Aligned BCW^a^ intervention function
Education	Provide information about heart failure. Provide information about self-care (eg, why you need to perform self-care activities and how to perform self-care activities). Improve health literacy. Provide training on problem-solving skills (eg, to respond to changes in symptoms or monitoring outputs). Provide information about how the intervention is beneficial. Provide staged information delivery over time.	Education, training, enablement, and persuasion
Monitoring and feedback	Monitor health (including physiological parameters and psychological status). Monitor self-care behaviors. Present data to the user, including that from medical investigations. Analyze trends and alert user (and health care practitioner) to changes outside of specified ranges. Provide feedback on health status changes.	Education, persuasion, environmental restructuring, and enablement
Social connection and support	Provide information about local groups relevant to heart failure and self-care activities. Connect users with other people with heart failure in the local area (like LinkedIn). Allow users to exchange stories and tips. Enable users to contact family, friends, and health care providers. Provide contact details for support.	Education, incentivization, environmental restructuring, and modeling
Psychological and emotional support	Provide tools for psychological well-being (including mindfulness). Provide support to change habits. Provide tools to address fear after diagnosis. Provide tools to help maintain sense of identity beyond health condition.	Education, training, and enablement
Planning and preparing	Provide a detailed care plan. Provide detailed instructions on how to perform self-care activities as they are being performed (eg, steps in taking medication). Tailor daily schedule to exercise tolerance. Remind the user to plan and perform self-care activities. Provide a place to record notes (eg, what was consumed). Provide access to a range of options along with decision-making support (eg, scan food for nutrition and database of common meals or foods). Deliver professionally approved advice and programs. Provide tools to train cognitive function (eg, memory games).	Education, training, environmental restructuring, and enablement
Health care support	Provide access to and communication with health care practitioners. Provide changes to the care plan when required. Support preparation for health care appointments (eg, notes and questions to cover in appointment). Share information with health care practitioner in advance of appointments. Support appointment booking. Share and store visual information during remote consultations. Provide information about different services and health care practitioners to support informed decision-making.	Education, environmental restructuring, and enablement

a BCW: Behavior Change Wheel.

The 6 intervention component themes were education, monitoring and feedback, social connection and support, psychological and emotional support, planning and preparing, and health care support. Each theme contains requirements derived from participants’ ideas. For example, in workshop 1, participants discussed a “marketplace” of health care professionals, so that they could make an informed decision about who to consult. In workshop 3, participants highlighted the ability to share their own notes and data with a health care professional prior to their appointment. These ideas were grouped together, forming part of the “health care support” theme.

Each component theme was mapped to relevant BCW intervention functions. Together, these 6 themes aligned with 7 of the 9 BCW intervention functions: education, persuasion, incentivization, training, environmental restructuring, modeling, and enablement. This mapping supports the theoretical grounding of the intervention and illustrates how the identified components could address the specific barriers to self-care identified earlier in the analysis.

#### User Requirements (“How” Requirements)

Beyond the intervention components (“what” requirements), the analysis of participants’ ideas for the intervention also generated 6 themes describing user requirements—“how” they wanted the intervention to function or operate. Table 3 shows each user requirement theme, along with specific participant-derived requirements.

**Table 3.** User requirements for a digital health self-care intervention for heart failure: requirements derived from workshops with people with heart failure (n=16) and caregivers (n=4) in Australia.

User requirement theme	Detailed user requirements derived from participants’ ideas
Physical design	Devices must be portable so that they can be used both in and outside of the home. Devices must have “friendly” esthetics and not look like medical devices. Monitoring devices must provide reliable and valid information. The number of devices should be kept to a minimum (ideally one). Devices should be compatible with those already owned.
Accessibility and usability	Information and data should be readable and accessible. Data should be presented simply (eg, use icons such as ticks or red, amber, and green). Language should be simple and avoid technical jargon. Data entry should be simple and upload automatically. Passive data collection options should be available. Data presentation should include support to interpret it. Devices must be easy to use. Devices should be preconfigured.
Personalization and control	Information should be tailored to health status, health literacy, and user preferences. Users should have the option to turn off unwanted features, alarms, or reminders. Users should have the option to customize alarms (eg, sounds, vibration, timing, and snooze). Users should have the option to rewind and pause videos. Users should have the option to control the visibility of information to users and the sharing of information with others.
Engagement and user experience	Users should be engaged using incentives and gamification (eg, digital badges and rewards). Users should be able to set health goals and share achievements with other users. Language and images should be inclusive and tailored to the user. Data should be presented in a meaningful way (eg, a digital twin). Information should be delivered in a staged approach. Follow-up educational support should be provided after initial learning. Education should be continued beyond initial diagnosis.
Support and implementation	The intervention should be provided soon after diagnosis or hospitalization. Support for technology setup and system orientation. Access to medical advice should be provided out of hours (eg, 24/7 messaging or helpline). Feedback on monitoring should be timely. Implementation should be delivered in a staged approach (ie, introduce intervention progressively). All health care practitioners should be involved. Caregivers should be involved. Human contact should be maintained. The intervention should be free or low cost.
Integration and system organization	The intervention should be integrated into the wider health care system to ensure joined-up care. The intervention should increase public awareness of heart failure.

The 6 user requirement themes were physical design, accessibility and usability, personalization and control, engagement and user experience, support and implementation, and integration and system organization. These themes reflect participants’ expectations for how the intervention should be delivered, designed, and supported in real-world use. For example, in workshop 2, participants discussed gamification techniques, such as stickers, stars, and rewards. In workshop 4, participants spoke about a desire to be able to share health achievements with others. These ideas were grouped together, forming part of the “engagement and motivation theme.”

### Integrating the Findings

Integrating the user needs (derived from self-care barriers) with the intervention components (what the intervention should include) and user requirements (how the intervention should operate) forms actionable insights that can be used to ideate solutions. For example, people with heart failure need knowledge and understanding of heart failure (user need); therefore, the intervention should provide education about heart failure (intervention component), and information should be readable and accessible and use simple language and avoid technical jargon (user requirements).

## Discussion

### Principal Findings

This study used a combined approach of the BCW and UCD to identify barriers to heart failure self-care and define actionable intervention requirements. By involving people with heart failure and caregivers across 4 workshops, we designed a comprehensive set of user needs, intervention components (“what” the intervention should include), and user requirements (“how” the intervention should function).

### Comparison With Prior Work

Some intervention components identified in this study reflect features of existing digital health interventions [16]. For example, one intervention improved self-care and quality of life, reduced hospital admission length, and included a home-based program with a tablet computer and wireless weight scale. It delivered educational modules, guideline-based adaptive diuretic titration based on weight changes, and alerts to contact a health care professional if signs of decompensation were detected [52]. These features align with the monitoring, education, and care-planning components identified in this study.

This similarity provides credibility to our methodology and findings, but it also raises questions about whether participants were limited by the boundaries of their knowledge and experiences with current technology and provision of self-care support. This is a recognized phenomenon in UCD [53]. Despite participants being encouraged to “think outside the box,” few of the suggested ideas were technologically advanced, and many were modeled off concepts found in social media platforms. This emphasizes the value of treating participants’ ideas as an intermediary step between understanding the users and designing solutions. Multidisciplinary teams, including the users in a user-centered co-design process, can then build upon these requirements to ideate novel solutions.

To illustrate, some participants reported avoiding daily weighing because it was a constant reminder of their illness, despite understanding its importance. Within the COM-B model, this reflects a motivational barrier; in such cases, interventions that simply persuade individuals to weigh themselves may be ineffective [26]. However, by acknowledging user requirements—such as enabling monitoring, simplifying self-care tasks, and reducing negative emotions caused by self-care—designers can explore alternative solutions. For example, noninvasive methods, such as speech analysis to recognize fluid overload [54] or a smart scale placed under a floor tile so that it goes undetected [55], could meet the same self-care goals while improving acceptability.

Furthermore, participant feedback is often shaped by their current context, without having interacted with the new intervention [53]. As a result, their ideas may theoretically meet their behavior change needs but may fall short once the intervention is implemented. For example, one intervention that used a touch screen computer to record health information and provide feedback on activity was found to be too large for participants’ homes, limiting its adoption [56]. Toward mitigating this, our approach followed an iterative process whereby participants could reflect on their ideas and provide additional thoughts for how they should be delivered. This approach may enhance early prototypes and reduce later costs, although pilot testing and further refinement are still necessary [37,57].

Some requirements identified in this study may appear to conflict. For example, one user requirement was that the intervention must be simple to use, while another emphasized the need for comprehensive features—this is an apparent tension, as added functionality can increase complexity. However, the capabilities of digital health technologies may provide an opportunity to strike a balance. Solutions such as modular interventions, where users can select only the components most relevant to them, or adaptive systems that tailor support based on user profiles (eg, health literacy or likes and dislikes), can help reconcile these tensions. As opposed to being obstacles, these tensions can provide stimulation for ideation.

### Strengths and Limitations

The strengths of this study lie in its application of the BCW and UCD methods. The study actively involved people with heart failure, ensuring that the requirements elicited were relevant to their needs. Participants were from both metropolitan and regional locations, which may have increased the diversity of experiences and ideas gathered in this study. Collecting and implementing feedback after each workshop meant that data collection improved as the study progressed. Furthermore, participants enjoyed contributing to the workshop, especially sharing their experiences with others, evidencing the inclusive environment generated and reinforcing the requirement for making connections with others [58].

The limitations of this study were identified as follows. The recruitment strategy for the workshops may have introduced a selection bias by using nurses to select potential participants [59]. However, an effort was made to diversify the participants by asking nurses to target a range of characteristics. Additionally, the participants who did participate in the workshops were likely to have a better health status and be more engaged in self-care as participating in a study requires a level of motivation and health. Notably, 3 participants could not attend their scheduled workshop because they were unwell, indicating that the findings may not be generalizable to a broader population, or transferable to a population with a worse health status [60]. Despite the strengths of the data collection methods, it is worth noting the potential limitations of the workshops. First, participants may have been less willing to share ideas or influenced by groupthink [61] in a workshop setting. We encouraged participants to write down their ideas if they preferred. To further overcome this, real-time feedback tools, such as interactive polls or digital platforms that allow participants to submit anonymous thoughts during the session, could be incorporated. Second, participants may have needed more time to think about their ideas. Providing information before the workshop and having a preliminary discussion in workshops 2 and 3 sought to minimize this limitation. It may have been of additional value to conduct multiple workshops, a more detailed follow-up survey, or a one-to-one interview with participants to ascertain further thoughts and ideas.

### Future Research

Building on the requirements identified in this study, frameworks such as the Integrate, Design, Assess, and Share (IDEAS) framework recommend ideating, prototyping, building, and testing the intervention [37]. It is also essential to incorporate perspectives from other stakeholder groups who will interact with the intervention or its outputs, including caregivers who had only minimal participation in this study and health care practitioners. Given that these groups may have conflicting priorities [54], a structured consensus-building approach, such as the Delphi survey methodology, may be valuable. This has previously been used with a group of health care practitioners to determine recommended features for smart technologies in heart failure care [62,63]. Continued involvement of people with heart failure is warranted to ensure that the intervention remains aligned with their needs. Further research should explore how to optimize involvement strategies to promote longer-term engagement.

### Conclusions

This study combined UCD and BCW to identify self-care barriers and define theory-based, user-centered requirements for a digital intervention to support people with heart failure. Our findings demonstrate how user-centered methods can augment the theory-based intervention development process of the BCW by generating actionable requirements that bridge a gap between understanding behavior and designing solutions. While existing interventions target self-care barriers regarding capability, opportunity, and motivation of people with heart failure, few address how these interventions should function in ways that are acceptable to users. This research provided the foundation for the design of a digital self-care intervention [36]. Future research can ideate and build solutions that meet the requirements outlined in this study and then test the feasibility, acceptability, and potential effectiveness of the resulting intervention.

## Supplementary material

10.2196/72589 Multimedia Appendix 1 Behavioral diagnosis.

## Abbreviations

BCW: Behavior Change Wheel
COM-B: capability, opportunity, motivation, and behavior
TDF: Theoretical Domains Framework
UCD: user-centered design
