# Supplementary material for: Developing Requirements for a Digital Self-Care Intervention for Adults With Heart Failure: Qualitative Workshop Study
Source: J Med Internet Res. 2025 Aug 25;27:e72589. doi: 10.2196/72589 (PMC12377876; doi:10.2196/72589)
Supplement: Multimedia Appendix 1 [file jmir-v27-e72589-s001.docx]

# Multimedia Appendix 1: Behavioral diagnosis

| COM-B | TDF | Barriers to performing the target behavior: self-care | The user needs (to)… | Illustrative quotes |
| --- | --- | --- | --- | --- |
| Physical capability | Skills | - Inability to perform self-care tasks due to heart failure-related symptoms, comorbidities, or side-effects. | - Address heart failure and comorbidity related symptoms and side-effects that affect physical ability to self-care. | *There's fluid restrictions but I get thirsty (Online)*  *Fitness levels – can’t keep up with others (W2)*  *It was hard and there were other health problems that came along because of the lack of irrigation, there were no tears or eye lubrications (W4)* |
| Psychological capability | Skills | - Difficulty in interpreting symptoms and attributing their causes. - Challenges in communicating health concerns with a healthcare practitioner. | - Interpret symptoms and their causes. - Communicate health concerns with healthcare practitioners. | *Not a good record keeper (W1)*  *I do it, I don’t interpret it, I give it to the doctors to determine where I’m at and what I need to do (W4)* |
|  | Knowledge | - Insufficient knowledge about heart failure and the importance of self-care. - Insufficient knowledge of resources and services available for support. - Inadequate understanding of self-care procedures. | - Knowledge and understanding of heart failure and the importance of self-care. - Knowledge of the resources and services available to support self-care. - Understanding of self-care procedures. | *Heart failure patients tell me that they’re handed medication forms to fill out, but the time isn’t taken to explain why they need to…weigh themselves each day, why they need to record the amount of fluid in & out (W4)*  *Weight fluctuations - What to do? (W2)*  *Not knowing what medications are for (W2)*  *Hardest thing was coming out – “what do we do now?” What happens? Are we meant to be watching her? (Online)* |
|  | Memory, attention, and decision processes | - Forgetting to perform self-care tasks (e.g., forgetting to take medication) due to cognitive impairment or distractions. - Forgetting how to perform self-care tasks (e.g., forgetting the medication dose) due to complexity of the task overwhelming cognitive ability. - Difficulty in making informed decisions about self-care actions. | - Retain/recall/remember information. - Remember self-care routines and tasks. - Focusing on all aspects of self-care - Make informed self-care decisions | *Difficult to remember what I’ve done and report back to healthcare practitioners (W2)*  *Couldn’t remember tablets, family coming in can then forget to take medication (W3)*  *Have to write things down, memory declines (W3)* |
|  | Behavioral regulation | - Difficulty with planning self-care tasks in advance. - Procrastination. - Difficulty in monitoring and regulating self-care behaviors. - Established habits oppose self-care. - Experiencing unexpected life events that hinder self-care (e.g. divorce). | - Planning self-care tasks in advance. - Monitor and adjust self-care behaviors. - Change opposing habits. | *Doesn’t fit into daily routine (Online)*  *Different source of fluid - hard to keep track (W2)*  *Very hard getting used to the fluid restriction and understanding that it’s not just liquid, its watermelon, grapes, anything (W2)* |
| Physical opportunity | Environmental context and resources | - Having a busy lifestyle and inconsistent routine. - Limited access to resources (e.g., low salt food, valid and reliable monitoring equipment, outdoor space, healthcare practitioners) – both generally and when away from home. - Receiving vague or contradictory advice from forums or healthcare practitioners. - Diverse external environmental conditions affect the ease or motivation to perform self-care. | - Access to the necessary resources for self-care. - Ensuring a conducive environment for self-care. - Perform self-care outside of the home. - Access/effective communication channels to healthcare practitioners for self-care support. - Clear and consistent advice on self-care. - Adapt to environmental changes. | *Weigh everyday – weight varies, I’ve been told – if my weight varies by 2kgs get in touch with doctor but it’s impossible to get in touch with doctor (W2)*  *They know what they’re talking about, we don’t – they try to explain as best as they can but you walk out confused (W4)*  *I had conflicting information – different teams of doctors were telling me different things (W4)*  *Organized exercise groups are good, but they end after 8 weeks, and they’re limited in small towns (W4)*  *Weather/seasonal changes (cold – no physical activity, warm – fluid restriction is difficult) (Online)* |
| Social opportunity | Social influences | - Social pressures encourage behaviors that oppose self-care. - Dependence on others to assist with self-care. - Lack of peers with heart failure for support and understanding. | - Independence or assistance with self-care depending on status - Address social pressures. - Connection with others/peer with heart failure for support and understanding | *Not sure where to find other people with heart failure, feeling very isolated (W2)*  *I found it hugely restrictive, you had to learn to say no – say no to every social cup of coffee (W4)* |
| Reflective motivation | Social/professional role and identity | - Self-care conflicts with social identity. | - Align self-care with personal and social identity | *I’m not going to call and bother people unless something is really really wrong (W2)*  *Also a lot of our food naturally is like curries and rice and they have a lot of water and a lot of proteins from dhals and that sort of stuff that you can’t have a lot of when you’re on a very strict diet that’s water based. And that was a struggle at the beginning (Online)* |
|  | Beliefs about capabilities | - Lacking confidence in ability to perform self-care. - Feeling disempowered about self-care tasks. | - Have the confidence to perform self-care. - Feel in control/ informed/empowered | *Feeling like you have lost control when pharmacist organizes medication (W2)*  *It’s good to have an active role in own care but data should be interpreted by people who understand it well. You worry yourself. (W4)* |
|  | Intentions | - Absence of intent to engage in some self-care tasks. | - Intent and motivation for self-care | *Most of my fluid now is because I love water. So I have to have my bottle of water and a cup of tea in the morning, and I have my beer at night, I’m a happy camper that way (Online)* |
|  | Goals | - Prioritizing other goals above self-care. | - Align self-care with other priorities | *I did so many things I shouldn’t have done (Online)* |
| Automatic motivation | Emotion | - Negative emotional responses to self-care cause reluctance or avoidance of self-care. | - Reduce the emotional consequences of self-care | *Doesn’t want to use scales because seeing the number reminds her that she’s unwell/not as fit as she used to be (W2, W4)*  *Weighing is a reminder of being unwell (W2)*  *I couldn’t relate to the information, and it sent me on a downward, depressive spiral (W4)*  *When I was first diagnosed – I was still trying to deal with ‘life threatening condition’ and then I was being told about fluid restriction (W4)* |
